# Comparison of *Albizia Julibressin* and Silver Sulfadiazine in Healing of Second and Third Degree Burns

**Published:** 2018-01

**Authors:** Hossein Asgarirad, Aroona Chabra, Mahnaz Rahimnejad, Ahmad Zaghi Hosseinzadeh, Ali Davoodi, Mohammad Azadbakht

**Affiliations:** 1 Department of Pharmaceutics, Faculty of Pharmacy, Mazandaran University of Medical Sciences, Sari, Iran; 2 Student Research Committee, Faculty of Pharmacy, Mazandaran University of Medical Sciences, Sari, Iran; 3 Department of Pharmacognosy, Faculty of Pharmacy, Mazandaran University of Medical Sciences, Sari, Iran; 4 Department of Plastic Surgery, Faculty of Medicine, Mazandaran University of Medical Sciences, Sari, Iran

**Keywords:** Herbal Medicine, Burns, Wound, Healing, Albizia julibressin

## Abstract

**BACKGROUND:**

Large numbers of population suffer from burn annually. The promising treatment of burn has not been identified yet. *Albizia julibressin* (*A. julibressin*) in *Fabaceae* family is popular for its antiseptic activity. This prospective study was designed to compare the wound healing effects of *A. julibressin* gel (AG) with silver sulfadiazine (SSD).

**METHODS:**

This single blind clinical trial was performed on 40 patients with second and third degree burns. 20 patients treated with SSD and 20 other patients received *A. julibressin*. The percentage of the wound healing was evaluated with pain, irritation, edema, itching, erythema, purulent discharges and skin discoloration symptoms. Also, the patients’ satisfaction and adverse drug reactions were determined.

**RESULTS:**

The severity of pain (*p*=0.03), inflammation (*p*=0.02) and purulent secretions (*p*=0.03) were significantly relieved in *A. julibressin* group. The healing time significantly reduced in second degree burns (*p*=0.03) and third degree burns (*p*=0.04) with treating by *A. julibressin*. No significant adverse drug reactions were detected with *A. julibressin*.

**CONCLUSION:**

It seems that *A. julibressin* improves the different therapeutic aspects of burn injuries and could be considered as a new herbal remedy in wound healings.

## INTRODUCTION

Skin and mucus membrane wound injuries might be caused due to several factors such as mechanical pressure and abrasions, trauma, animal or insect bites, burns, etc. Burn is a major pathologic condition with high prevalence that can occur among all genders in all developed and developing societies and actually is a global life threating disorder inducing morbidity and mortality.^1^^,^^2^ In addition, as thermal burn is associated with scarring, so the healing process should decrease the scar and its related problems.^3^^,^^4^

Actually, healing of burn wounds and scars has a complexity, because burn results into infection, inflammation, remodeling and granulation of the tissue and organ.^5^ There are many chemical and herbal topical drugs used for the burn treatment.^1^^-^^8^ In all traditional medicines, plants are widely used for healing of the burn wounds. In addition, Iranian traditional medicine has suggested a lot of plants for healing of burn wounds.^7^^,^^9^

**Fig. 1:**
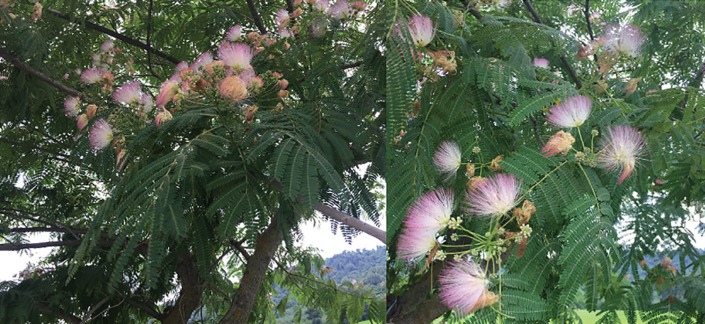
*A. julibressin* tree in Hizarjarib forest, near Neka city, Mazandaran Province, Iran.

*Albizia julibressin *Durazz (Mimosa, Persian silk tree) from *Fabaceae* family was described as an appropriate healing agent in Iranian traditional medicine.^10^ *A. julibressin *is actually a plant with high trees (up to 6 meters), short trunk, a broad crown and pink flowers (Figure 1). The plant was distributed in the tropical America, Africa and Southeast Asia. In addition, this plant was cultivated in other countries too.^11^ This plant is used in traditional medicine for inflammation and insomnia treatment. In pharmacology studies, Albizia showed antitumor activity and anti-platelet effects.^12^^,^^13^ Literature has revealed important biological activities for crude extracts and isolated and purified substances of some species of Albizia including anticonvulsant, sedative, anti-inflammatory, antitumor and antimicrobial activity.^14^

The main compounds** t**hat are isolated from Albizia genus are triterpenoid saponins.^15^ Saponins are secondary metabolites widely distributed in the plants.^12^ These natural compounds have wide range of pharmacological and therapeutically properties such as expectorant, anti-inflammatory, immunomodulatory, hypoglycemic, antifungal, antiparasitic, etc.^15^ This study has focused on the wound healing properties of *A. julibressin *in burn wound ijuries and compared its effect with topical silver sulfadiazine (SSD) in a clinical trial.

## MATERIALS AND METHODS

The stem’s bark of *A. julibressin *was collected from Hizarjarib forest, near Neka city, Mazandaran Province in north of Iran. In addition, herbarium sample of the plant was prepared. Taxonomic identification of the plant samples was performed by Professor Mohammad Azadbakht, and representative voucher specimens were deposited at the herbarium site of the Department of Pharmacognosy, Faculty of Pharmacy, Mazandaran University of Medical Science, Sari, Iran. The samples were washed and dried in an oven at 40°C for 4 h. 

The dried bark was powdered and extracted by ethanol (60%) using digestion method and was concentrated under a vacuum and freeze-dryer.^7^ A total of 13.18 g of dried extract was achieved from 100 g of the dried bark powder. The total triterpenoid saponins as an active ingredient in Albizia stem’s bark was assayed by UV spectrophotometric method based on Aescin amount. Ten grams of dried extract was dispersed in 25 mL FeCl3/acetate solution (DAB 2002) and heated in 60^o^C for 25 minutes. Then, the final solution was evaluated in 540 nm by appropriate blank solution. Calibration curve was plotted by Aescin standard solutions.^16^ Total amounts of saponin were assayed before and after preparation of the *A. julibressin*. All chemicals such as standard of Aescin, the gel ingredients and solvents were purchased from Merck Company, Germany. 

The topical 5% *A. julibressin* was prepared by dissolving 5 g of dried extract and methyl (0.18 g) and propyl paraben (0.02 g) as preservatives in water (the total volume of solution was 100 ml). Twenty grams of carbopol polymer (type 940) and 20 g of glycerin were dispersed in the solution overnight. The solution was shacked and several drops of triethanol amine (TEA) were added until gel formation. The best formulation was 5% herbal extract, 0.2 preservative, 1% carbopol, 5% glycerine, 88.3% water and 0.5% triethanolamine. 

The final pH of *A. julibressin *was 5.2. Physicochemical evaluation of topical *A. julibressin* including pH, sineres and stability were performed according to the United States Pharmacopeia (USP) monograph and microbial quality control using Soybean-Casein Digest Medium (SCDM) culture environment.^17^ According to the Beer-Lambert law, the total amount of triterpenoids saponins of extract and gel based on Aecsin in absorbance of 0.075 and 0.099 was 24.74% and 1.92%, respectively. The products maintained their physical stability at 4, 25 and 40 °C during the last three months. Microbial limit tests were performed to determine the absence of *Candida albicans*, *Pseudomona auroginosa* and *Staphylococcus aureus*, and other aerobic, anaerobic and pathogenic micro-organisms in the products. All the steps of the experiment were repeated three times.

This prospective, randomized and double-blinded clinical trial was carried out after the Ethical Committee approval at Mazandaran University of Medical Sciences and was performed on 40 patients with second-degree burns who were admitted to the burn emergency ward at Zare Hospital in Sari City, Mazandaran Province. The consent forms were signed by all patients. The patients with ages of 20–60 years, non-pregnant, non-diabetic, non-epilepsic and non-sensitive to herbal extracts were considered as including criteria. All patients were randomly divided equally in two groups in which 20 patients received 5% *A. julibressin *and 20 remained patients received 1% SSD.

The clinical parameters such as inflammation, pain, edema, itching, erythema, purulent discharges and skin discoloration were compared between two groups.^7^^,^^10^ First after admission, patients were evaluated by an expert emergency burn physician. The wounds were washed with normal saline and bandage with SSD or *A. julibressin*, daily. All patients were treated under the same condition and nutrition. The clinical parameters were followed up by the physician on 1, 3, 5, 7, 10, 13, 15, 20, 25, and 30 days of the burn injury. In addition, the wound condition and healing time were recorded. Quantitative and qualitative data were analyzed by t-test and Chi–Square test, respectively using SPSS software (Version 21, Chicago, IL, USA). A p value<0.05 was considered as statistically significant. 

## RESULTS

Forty qualified patients with second and third degree burns at Zare Burn Hospital with their logical consent were involved in the study. None of the patients left the study and all of them were followed up until the end of study. As shown in Table 1, the average patients’ age was 33.5±1.4 and 35.5±1.6 years in SSD and AG groups, respectively, without any statistically significant difference between the two groups (*p*=0.52).

**Table 1:** The demographic characteristics of patients in the clinical trial.

**Variables**		**Silver sulfadiazine** **No. (%)**	***A. julibrissin*** **No. (%)**	***p *** **value**
Age (Mean±SD, Years)		35.5±1.6	33.5±1.4	0.52
Gender	Female	11 (55)	15 (75)	0.39
	Male	5 (25)	9 (45)	
Place of living	City	11 (55)	11 (55)	0.44
	Village	9 (45)	9 (45)	
Area	Upper limb	9 (45)	7 (35)	0.86
	Lower limb	8 (40)	8 (40)	
	Trunk	3 (15)	5 (25)	
Degree	Second	12 (60)	10 (50)	0.71
	Third	8	10	

Women constituted the most part of the population in the study (65%) however, gender had no significant effect on wound healings (*p*=0.39). Fifty five percent of patients were living in the city otherwise, 45% in villages with no significant difference (*p*=0.44). Majority of the burns in both groups (70% in SSD and 75% in *A. julibressin* groups) occurred by hot liquids especially boiling water and affected a range of 1-5% of burns. The locations of burn surface areas were upper limb (40%), lower limb (40%) and trunk (20%) with no significant difference between the two groups (*p*=0.86). Fifty five percent of patients had second and 45% suffered from third degree burns with no significant difference between the two groups (*p*=0.71). 

**Fig. 2:**
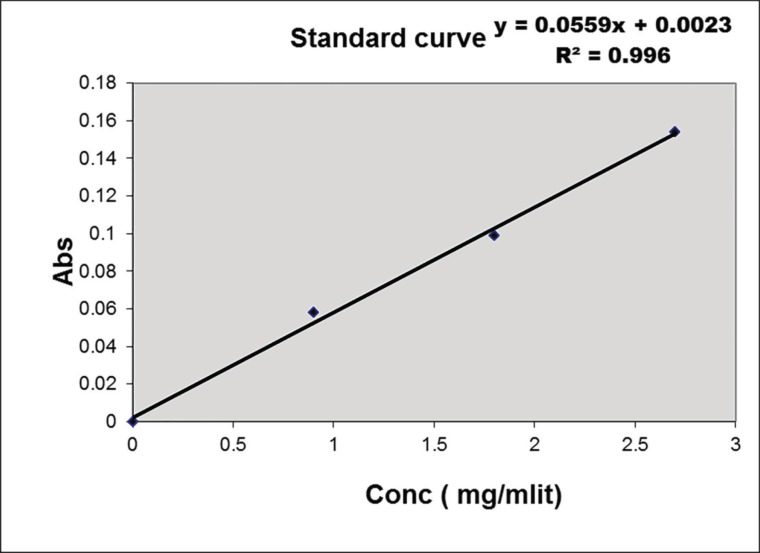
Standard curve of the total amount of triterpenoids saponins based on Aecsine.

**Fig. 3:**
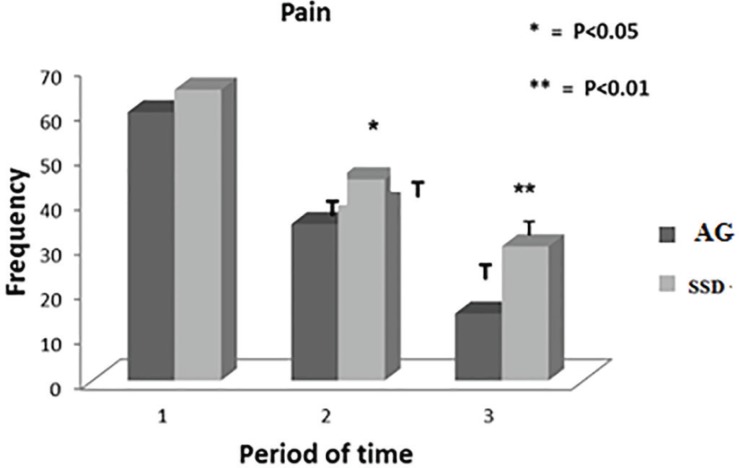
Comparison of relieving pain in *A. julibrissin* (AG) and silver sulfadiazine (SSD) groups (1: first of the admission, 2: The middle day of treatment period, 3: The last day of treatment).

**Fig. 4:**
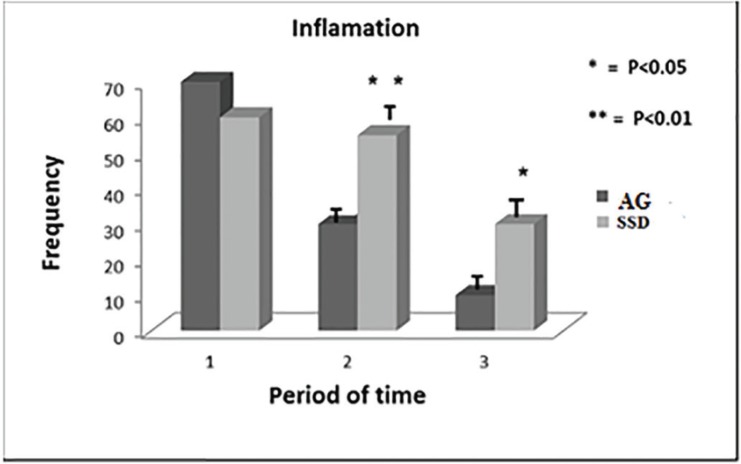
Comparison of relieving inflammation in *A. julibrissin* (AG) and silver sulfadiazine (SSD) groups (1: first of the admission, 2: The middle day of treatment period, 3: The last day of treatment).

The daily clinical examinations showed significant wound healing progress in both groups. There were no significant differences in the clinical symptoms of all patients at the first of the admission before any medical intervention (Table 2). The comparisons of patients’ clinical symptoms improvement at the middle and end of the periods were shown in Table 3 and 4. In treatment group consuming *A. julibressin*, a significant reduction was noticed for pain (*p*=0.039 at the middle of the period, *p*=0.03 at the end of the period), inflammation (*p*=0.04 at the middle of the period, *p*=0.02 at the end of the period), purulent secretions (*p*=0.03 at the middle of the period, not significant at the end of the period) in comparison to control group following consuming SSD (Figures 2, 3 and 4). 

**Table 2:** The comparison of patients’ clinical signs between two groups at the first of the admission.

**Clinical signs**		**Silver sulfadiazine** **No. (%)**	***A. julibrissin*** ** No. (%)**	***p *** **value**
Inflammation	Severe	12 60)	14 (70)	0.85
	Moderate	6 (30)	3 (15)	
	Mild	2 (10)	3 (15)	
Pain	Severe	13 (65)	12 (60)	0.77
	Moderate	4 (20)	5 (25)	
	Mild	3 (15)	3 (15)	
Itching	Severe	1 (5)	2 (10)	0.12
	Moderate	9 (45)	7 (35)	
	Slight	10 (50)	11 (55)	
Erythema	Severe	6 (30)	8 (40)	0.09
	Moderate	10 (50)	7 (35)	
	Mild	4 (20)	5 (25)	
Edema	Severe	4 (20)	6 (30)	0.25
	Moderate	3 (15)	3 (15)	
	Mild	13 (65)	11 (55)	
Purulent discharge	Severe	9 (45)	8 (40)	0.76
	Moderate	8 (40)	9 (45)	
	Mild	3 (15)	3 (15)	
Textures color change	Severe	7 (35)	6 (30)	0.19
	Moderate	7 (35)	8 (40)	
	Mild	6 (30)	6 (30)	

**Table 3:** The comparison of patients’ clinical signs in two groups at the middle period of the treatment.

**Clinical signs**		**Silver sulfadiazine** **No. (%)**	***A. julibrissin*** ** No. (%)**	***p *** **value**
Inflammation	Severe	11 (55)	6 (30)	0.04
	Moderate	6 (30)	13 (65)	
	Mild	3 (15)	1 (5)	
Pain	Severe	9 (45)	7 (35)	0.04
	Moderate	10 (50)	6 (30)	
	Mild	1 (5)	6 (35)	
Itching	Severe	4 (20)	2 (10)	0.35
	Moderate	9 (45)	12 (60)	
	Slight	7 (35)	6 (30)	
Erythema	Severe	10 (50)	9 (45)	0.58
	Moderate	6 (30)	6 (35)	
	Mild	4 (20)	5 (25)	
Edema	Severe	2 (10)	1 (5)	0.64
	Moderate	6 (30)	8 (45)	
	Mild	12 (60)	11 (55)	
Purulent discharge	Severe	9 (45)	3 (15)	0.03
	Moderate	6 (30)	4 (20)	
	Mild	5 (25)	13 (65)	
Skin discoloration	Severe	5 (25)	6 (30)	0.06
	Moderate	7 (35)	7 (35)	
	Mild	8 (40)	7 (35)	

**Table 4:** The comparison of patients’ clinical signs in two groups at the end of the treatment.

**Clinical signs**		**Silver sulfadiazine** **No. (%)**	***A. julibrissin*** ** No. (%)**	***p *** **value**
Inflammation	Severe	6 (30)	2 (10)	0.02
	Moderate	10 (50)	8 (40)	
	Mild	4 (20)	10 (50)	
Pain	Severe	6 (30)	3 (15)	0.03
	Moderate	7 (35)	6 (30)	
	Mild	7 (35)	11 (55)	
Itching	Severe	5 (25)	2 (10)	0.51
	Moderate	12 (60)	16 (80)	
	Slight	3 (15)	2 (10)	
Erythema	Severe	6 (30)	4 (20)	0.44
	Moderate	9 (45)	9 (45)	
	Mild	5 (25)	7 (35)	
Edema	Severe	0	0	0.95
	Moderate	0	0	
	Mild	20 (100)	20 (100)	
Purulent discharge	Severe	0	0	0.84
	Moderate	0	0	
	Mild	20 (100)	20 (100)	
Textures color change	Severe	4 (20)	5 (25)	0.41
	Moderate	12 (60)	13 (65)	
	Mild	4 (20)	2 (10)	

In other symptoms including itching, erythema, edema, skin discoloration, a better therapeutic effect was observed in treatment group, but was not statistically significant (*p*>0.05). In treatment group, the average days of the re-epithelialization was significantly shorter than the control group for 2^nd^ degree burns (4.5±1.31 versus 6.75±1.54, *p*=0.03). A similar effect was seen for 3^rd^ degree burns (7.8±1.34 versus13.87±2.6, *p*=0.04, Table 5). 

**Table 5:** Compare of mean±SD time (days) of cure in placebo group and drug group.

**Burning degree**	**Silver sulfadiazine**	***A. julibrissin***	***p *** **value**
2^nd^ degree	6.75±1.54	4.5±1.31	0.03
3^rd^ degree	13.87±2.6	7.8±1.34	0.04

**Fig. 5:**
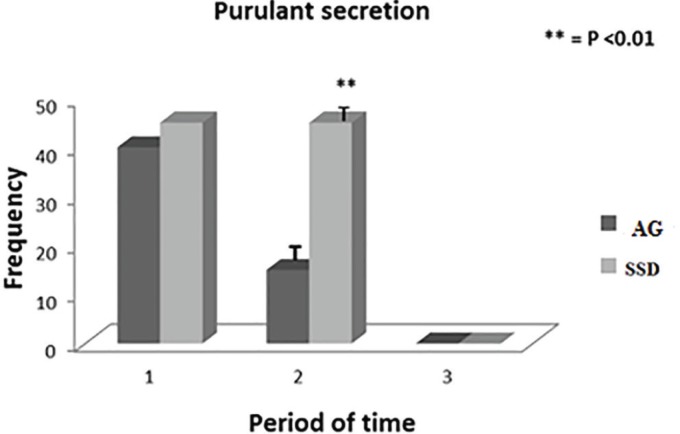
Comparison of relieving purulent discharge in *A. julibrissin* (AG) and silver sulfadiazine (SSD) groups (1: first of the admission, 2: The middle day of treatment period, 3: The last day of treatment).

**Fig. 6:**
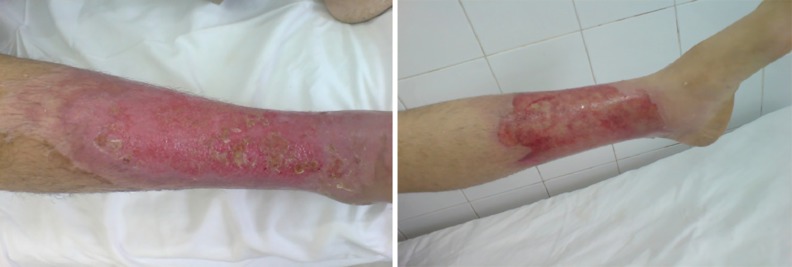
Comparison of the wound healing progress in third degree burns between two groups after 14 days of treatment (left: silver sulfadiazine, right: *A. julibrissin*).

**Fig. 7:**
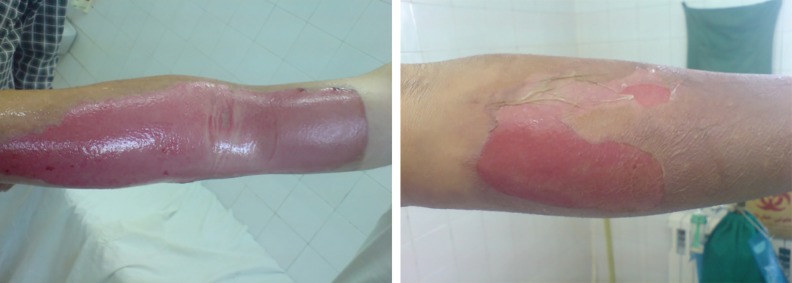
Comparison of the wound healing progress in second degree burns between two groups after 14 days of treatment (left: silver sulfadiazine, right: *A. julibrissin*).

Therefore, treating with *A. julibressin* decreased 33.3% of time period for treatment in second degree burns and 43.78% reduction in the duration time of therapy in the 3^rd^ degree burns (Figures 5). No adverse drug reaction or associated injuries was noted by consuming the *A. julibressin* during the treatment. In Table 6, the patients’ satisfaction with the product was reported, while 65% of the patients were unsatisfied for the color of *A. julibressin*, 15% for the odor and 10% for stability. But in SSD group, more than 50% of patients were satisfied with the product. Generally better therapeutic effects were achieved in *A. julibressin* group (Figure 6 and 7). 

**Table 6:** Patients satisfaction from product formulation.

**Satisfaction scale**	**Silver sulfadiazine: No. (%)**			***A. julibrissin*** **: No. (%)**			***p *** **value **
	**Color**	**Odor**	**Stability**	**Color**	**Odor**	**Stability**	
Excellent	12 (60)	10 (50)	14 (70)	2 (10)	6 (30)	11 (55)	<0.05
Good	5 (25)	5 (25)	5 (25)	5 (25)	11 (55)	7 (35)	
Moderate	3 (15)	5 (25)	1 (5)	13 (65)	3 (15)	2 (10)	

## DISCUSSION

In this clinical trial, the wound healing properties of *A. julibressin* on second and third degree burns have been investigated. In patients who were consuming *A. julibressin*, better effects were seen particularly in reliving pains, inflammation and time period of healing. The re-epithelialization took less time in *A. julibressin* group rather than SSD group. So we saved approximately 8 days of hospitalization. Reducing the hospitalization duration would benefit the government for the costs. Also, the hazard of infection and the need of prescribing associated drugs following adverse drug reactions would decrease. 

All patients did not show any complications such as infection, dermatitis or any other serious reactions. We could demonstrate equal safety with SSD and *A. julibressin *as was shown before.^18^ Products with antimicrobial properties are the drug of choice for prescription in burns due to their prophylactic action for reducing the risk of the infection.^19^ Also, the medicinal plants, those containing anti-inflammatory and antioxidant effects are more applicable for burns treatment.^20^

Phytochemical investigation of different species belonging to genus *Albizia *afforded different classes of secondary metabolites such as saponins, terpenes, alkaloids and flavonoids. Some bioactive compounds isolated and identified from genus *Albizia *were triterpenoid saponins (julibroside J29, julibroside J30, julibroside J31, induction of apoptosis), novel macrocyclic alkaloids (budmunchiamines A, B and C) and two flavonol glycosides (quercitrin and isoquercitrin) showing different biological activities such as antitumor, anti-inflammatory, antidiabetic, antiplatelets aggregation anti-oxidative, anti-viral and bactericidal functions.^21^^-^^23^

Saponin compounds have been proved to have anti-inflammatory activities in pain relieving, edema and skin inflammation induced by tissue injuries.^12^ The main wound healing of *A. julibressin* was possibly attributed to saponin compounds in which elevating the level of vascular endothelial growth factor and the inflammatory cytokines via exciting the fibroblasts, endothelial cells and macrophages to migration at wound sites to rebuilt the matrix and new tissue.^15^ The flavonoid components of *A. julibressin* react with superoxide radical anions which are produced by neutrophils and macrophages leading to reducing swelling and inflammation.^9^^,^^18^

Also the hydroxyl groups of flavonoids attack to the microbial cell membranes and inhibit the microbial infection.^20,22^ The high amounts of *A. julibressin* tannins have strong antioxidants activity to reduce the free radicals and to attach to the pathogens cell wall and astringent on wounds.^21-23 ^Rajalakshmi and Senthil (2014) exhibited alcoholic extracts of *A. julibrissin* with good inhibitory effects at concentration of 200 μg/ml on *Bacillus cereus*, *Escherichia coli*, *Enterococcus faecalis, Klebsiella pneumonia, Proteus vulgaris, Pseudomonas aeruginosa, Salmonella typhi, Salmonella paratyphi, Staphylococcus aureus, *and *Staphylococcus epidermis *and predicted *A. julibrissin* as a potent antiseptic agent too.^24^

Among the different events that are hazardous for human health or life, burn is the most heinous event that could suffer individuals or societies.^1^ Different studies have suggested herbal or non-herbal components on burn wound healing, some of them came to pharmaceutical markets; however, some made undesirable side effects or unprofitable healing action.^25^^,^^26^ Usually, wound dressing, amniotic membrane and potent antibacterial drugs are recommended for burns treatments. However, daily burn dressing may cause excessive costs and despite the positive amniotic membrane benefits to protect the injuries before surgery, it may lead to microbial contaminations. Also, the FDA approved antimicrobial drugs such as Sulfamylon, Nitrofurazone, Silver sulfadiazine, etc., however, they may cause tenderness or pain, perilous, biochemical changes and toxicity.^27^

Also, the antimicrobial drugs have resistance and limit of low penetration when used by prescriptions. For example, silver sulfadiazine as a broad-spectrum antibacterial drug, is the first choice in burn treatment. Whereas, the delayed wound healing as well as cell toxicities concern the physicians during the treatment procedures.^1^^-^^3^^,^^9^ The use of medicinal plants is increased in health care system over the years and has been considered for the treatment of various skin disorders and dermatologic diseases and especially for the cuts, burns and wounds from long time ago.^28^ Choosing the appropriate medicinal plants has financial profits and also they do not have any side effects in comparison with chemical drugs. 

In Iranian traditional medicine, one species in *Fabaceae* family including *Mimosa pudica* has been used for skin disorders or bandaging the skin scares, burns or psoriasis with raw *Mimosa teniuiflora (Tepescohuite)*.^29^ Similarly, in Mexico from past decades ago, using *Mimosa pudica *for curing the burn scares that had been caused by explosion was shown to have a good progress in remedy.^30^ Recent surveys conducted in France have shown the restorative power of *Mimosa* in cell fusion. Howbeit *A. julibrissin* is an endemic flora of Iran, but rare studies have been performed in our country. Kokane *et al.* revealed that phenolic compounds in the *A. julibrissin* extract to accelerate the wound healing process.^22^ The high amount of saponines and polyphenol compounds such as tannin in the plant make it as a promising anti-microbial and wound healing product.^31^^, ^^32^

In our investigation, *A. julibrissin *had a good curing effect on second and third degree burns. It is also better to measure the *A. julibrissin *wound healing on bedsore. The most patients’ dissatisfaction was recorded on product color (65%), that it is necessary to improve the appearance of the products. Our team recommended that, the effectiveness of different concentration of *A. julibrissin *must be evaluated and performed on larger size of populations. The product standardization for tannins and the expiration date are needed to be determined. The histochemical and cellular pathology assessments on wound injuries seem necessary too. 
